# A systematic review to characterize functional tic-like behaviors

**DOI:** 10.3389/frcha.2026.1936806

**Published:** 2026-10-02

**Authors:** Joseph B. Gary, Jacob S. Shaw, Joseph F. McGuire

**Affiliations:** 1 Department of Psychiatry and Behavioral Sciences, Johns Hopkins University School of Medicine, Baltimore, MD, United States; 2 Department of Psychiatry, Harvard Medical School, Cambridge, MA, United States

**Keywords:** comorbidity, functional neurological disorder, functional tic-like behavior, phenomenology, tourette syndrome

## Abstract

**Background:**

Functional tic-like behaviors (FTLBs) have emerged as a prominent clinical presentation within the context of the COVID-19 pandemic. Although FTLBs share phenomenological overlap with Tourette Syndrome and other tic disorders (collectively Tourette Syndrome, TS), evidence suggests that differences exist in demographics, phenomenology, clinical course, psychiatric comorbidity, and response to intervention. Given the rapid rise in the incidence of FTLBs—along with their complex clinical presentation—we provide a systematic review and integrated synthesis of the extant literature.

**Methods:**

We conducted a systematic review following PRISMA guidelines. PubMed and PsycINFO databases searches were completed in June 2026, using terms related to functional tics and TS. Inclusion criteria were English-language studies reporting clinical data on FTLB with sample sizes of 8 or greater. Case reports, non-human studies, and commentaries were excluded. Thirty-nine studies were included. Data were extracted on demographics, phenomenology, clinical course, psychiatric comorbidity, and treatment responsiveness. Since multiple reports originated from the same data repositories, information was synthesized qualitatively by study sample and conceptual domain.

**Results:**

The results of our review suggest that FTLBs predominantly affect adolescents and young adults and exhibit a strong female predominance—with higher than anticipated representation of gender-diverse individuals. Compared with TS, the onset of FTLB symptoms was abrupt and characterized by complex motor movements and vocalizations that cause functional impairment. While there is a considerable overlap between FTLBs and TS, there were distinctions in patterns of psychiatric comorbidities. Pharmacological and cognitive behavioral therapy (CBT) provided some benefit for individuals with FTLBs, but the therapeutic target of these interventions was not specified.

**Conclusions:**

While sharing some similarities, FTLBs have a distinct clinical presentation from TS. There is some evidence to inform treatment approaches, but findings are complicated by the retrospective nature of most extant reports. Future research is essential to characterize longitudinal trajectories, underlying mechanisms, and evaluation of targeted interventions in randomized clinical trials.

## Introduction

Historically, outbreaks of “hysteria” or “conversion disorders” are psychosomatic illnesses that have followed patterns of social contagion. While these patterns have been circumscribed to small localized social networks, technological innovations in communication (e.g., social media) have provided a larger platform for the spread of psychosomatic illnesses. For instance, in 2011–2012 there was a notable cluster of tic-like symptoms that suddenly emerged among 18 adolescents and one young adult at a high school in Le Roy, New York. These sudden-onset movements and vocalizations prompted public concern about the spread of a“mystery illness,” and the New York State Department of Health characterized the outbreak as mass psychogenic illness—with experts hypothesizing that intense media attention may have exacerbated these symptoms (1). The Le Roy event highlighted that tic-like symptoms can emerge and/or propagate in an abrupt manner and provided initial evidence of how social factors (e.g., media coverage) can contribute to an increase in symptoms.

In the context of the COVID-19 pandemic, clinicians across the world began reporting a marked increase of adolescents with an acute and explosive onset of tic-like behaviors (2, 3). In many cases, these patients did not initially report a prior history of tics, but presented with complex movements and/or vocalized outbursts that often resulted in visits to the emergency department (3–6). Interestingly, when exploring factors associated with these complex movements and vocalizations, a common theme emerged regarding the presence of increased psychosocial stressors—with some symptoms triggered and/or exacerbated by viewing social media related to others exhibiting these similar symptoms (e.g., TikTok, YouTube) (7). These functional tic-like behaviors (FTLBs) became a well-recognized clinical phenomenon that existed at the intersection between the broader categories of both Tourette Syndrome and functional neurological disorders (FNDs). Specifically, Tourette Syndrome and other persistent tic disorders (collectively Tourette Syndrome, TS) are characterized by the presence of motor and/or vocal tics that have a well-described onset and clinical course (8). The symptoms of TS onset in childhood, progress in a rostro-caudal pattern, and peak in tic severity during early adolescence (8). Evidence suggests that TS affects boys more than girls, with individuals often exhibiting comorbid conditions like attention deficit hyperactivity disorder (ADHD) and obsessive-compulsive disorder (OCD). Meanwhile, FNDs are characterized by neurological symptoms—such as weakness, abnormal movements, or seizure-like episodes—that are incongruent with known structural or neurodegenerative pathologies (9). These symptoms can have an abrupt onset and are believed to arise from abnormalities in functional connectivity, network dynamics, or attentional processing within the brain, rather than from identifiable lesions or neurochemical imbalances (9, 10). There are clear distinctions between TS and FNDs, but also some notable overlaps between the two conditions—as some patients with TS can also present with FTLBs (11, 12).

Although it can be challenging to differentiate between TS and FTLBs, it is important to understand the difference between these two conditions, as these conditions have different clinical courses, prognoses, and responses to interventions. Towards this goal, we conducted a systematic review of published reports to synthesize the extant information regarding demographic characteristics, phenomenology, clinical course, comorbid conditions, and response to interventions among individuals with FTLBs. Findings represent the first comprehensive review of the FTLB literature and will identify key gaps in the extant literature to guide future research.

## Methods

### Search strategy

A comprehensive literature review of FTLB following Preferred Reporting Items for Systematic Reviews and Meta-Analyses (PRISMA) guidelines was conducted. The search was supplemented by examining reference lists of review articles. PubMed and PsycINFO databases were initially searched on November 2024 and then updated on June 21, 2026, using the following search terms: (“tic” OR “tic disorder” OR “Tourette* Syndrome” OR “Tourette*” OR “involuntary movement” OR “non-epileptic motor phenomena” OR “non-epileptic motor events”) AND (“somatoform disorder” OR “conversion disorder” OR “functional neurologic disorder*” OR “functional movement disorder”) OR (“functional tic-like behavior” OR “psychogenic tic” OR “functional tic” OR “psychogenic movement disorder”). Titles and abstracts were screened for relevance. Full inclusion criteria were the following (1): reporting clinical data on FTLBs (e.g., case series, cohort studies, surveys, or qualitative studies). Exclusion criteria were the following (1): studies with fewer than 8 patients/participants, (2) studies not available in English, (3) non-human studies, and (4) articles that were reviews or commentaries. Two reviewers (J.G. and J.S.) independently assessed all eligible articles and references for inclusion. Consensus was achieved on the final set of articles for inclusion amongst the three authors (J.G., J.S., and J.M.)

### Study coding and data extraction

Studies were coded for the following information: (1) sample size, (2) demographic information (e.g., average age, percent female), (3) site/location, (4) clinical features of FTLB, (5) comorbid neurologic and psychiatric conditions, and (6) treatment findings. Given the heterogeneity of study designs, data were synthesized qualitatively. Given this is a systematic narrative review (and not quantitative review), a formal meta-analysis was not performed because several reports utilized the same clinical sample at different time points. Rather, findings were integrated thematically and by study site.

### Risk of bias assessment

The methodological quality of included studies was appraised using the Joanna Briggs Institute (JBI) Critical Appraisal Tools appropriate to each study design: case series (13), cross-sectional (14), quasi-experimental (15), cohort (16), and case-control (16). Studies were classified into design categories based on their primary analytic contribution rather than author self-classification. Rating percentages were calculated as the proportion of Yes responses among applicable items (excluding Not Applicable). Studies were categorized as Low (≤49% Yes), Moderate (50%–69% Yes), or High (≥70% Yes) methodological quality. All studies were appraised by one reviewer (J.G.). To assess inter-rater reliability, a stratified random sample of 10 studies (25.6% of the total sample) was independently dual-appraised by a second reviewer (J.S.) blinded to the first reviewer’s ratings. Across 96 paired item-level ratings, raw agreement was 85.4% and Cohen's *κ* = 0.66 (95% CI 0.49–0.82), indicating substantial agreement. The majority of disagreements (12/14, 86%) involved adjacent categories (Yes vs. Unclear or Unclear vs. No). All discrepancies were resolved through discussion between the two reviewers.

## Results

### Included studies

Search strategies produced 334 potential abstracts for initial review. Figure 1 presents the PRISMA Flow diagram that resulted in 39 studies included in the present systematic review. Table 1 presents narratively summarized study findings. Table 2 presents extracted data regarding patient characteristics, phenomenology, clinical course, comorbid psychiatric conditions, and response to interventions. Risk of bias assessment revealed substantial heterogeneity in methodological quality, with variation both within and across design categories (See Table 1). Several methodological patterns emerged: First, there was significant heterogeneity in the FTLB subtype(s) that studies included secondary, *de novo*, functional overlay, and social-media-associated presentations, or a combination. Second, existing literature is dominated by descriptive case series and cross-sectional group comparisons at single time points; no studies employed genuine longitudinal comparative cohort designs with both a comparator group and prospective outcome ascertainment*.* Third, most studies reported only univariate between-group comparisons without multivariable modeling. Fourth, comorbidity ascertainment across the FTLB literature typically relied on clinical assessment rather than standardized psychiatric instruments (e.g., PHQ-9, GAD-7). Finally, only two intervention studies were identified, both of which employed uncontrolled single-arm pre-post designs.

**Figure 1:**
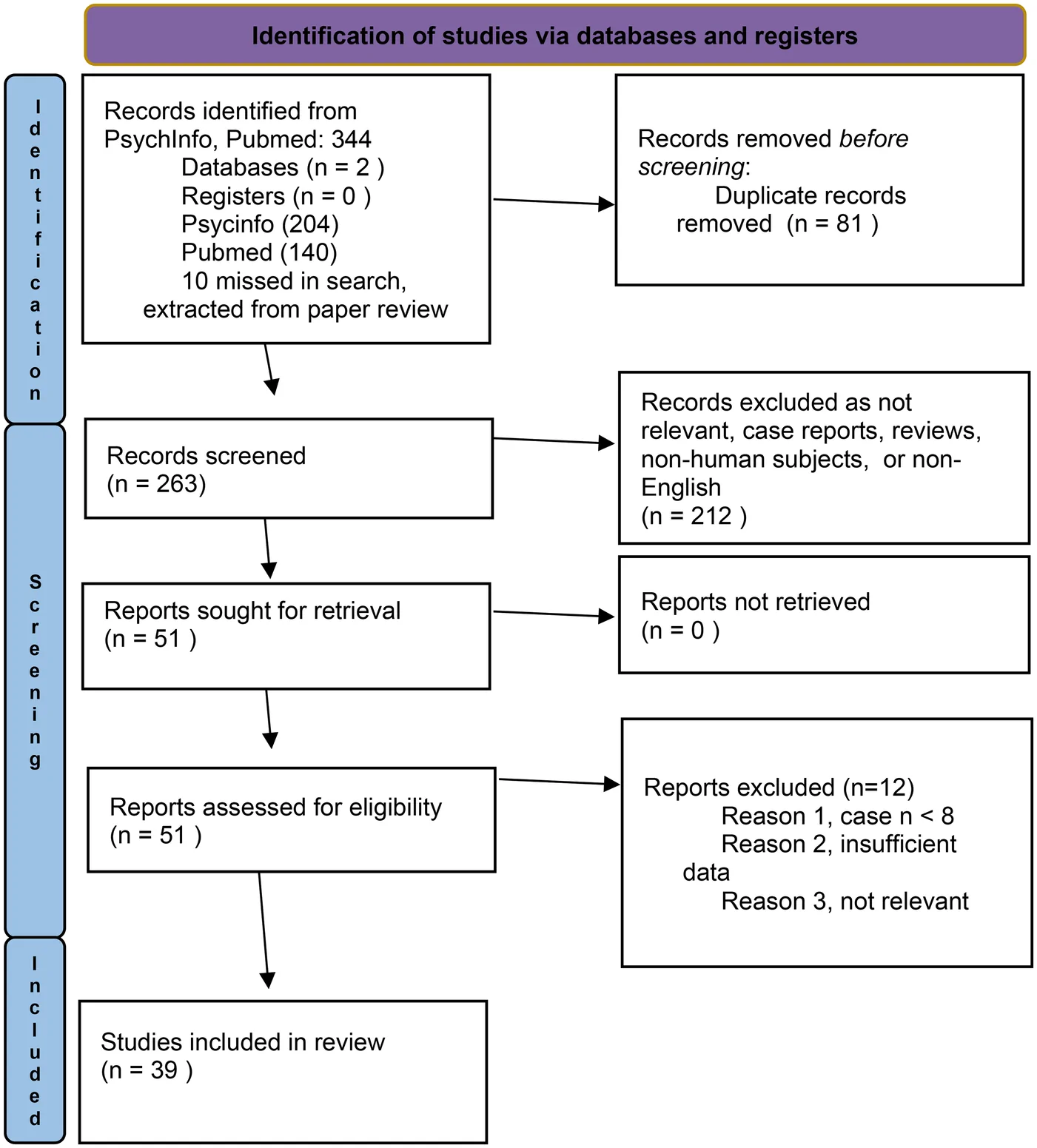
PRISMA flow diagram. Search conducted on Nov 11 2024 and updated on Jun 21 2026.

**Table 1:** Narrative summary by paper.

Authors (Year)—Paper	Study Type, location, date	Cohort Overlap?	Narrative Summary	Study Quality
Mejia & Jankovic (2005) (33)—Secondary tics and tourettism	Retrospective clinic series; single center (Baylor College of Medicine Movement Disorders Clinic, USA); 1981–2005		Retrospective adult movement disorder clinic series characterizing secondary/associated causes of tics, of whom 10.3% were classified as psychogenic tics. Precipitating stressors identified included medical/surgical procedures, workplace incidents, family death, leaving home, sports competition, motor vehicle accidents, acute panic attack, or meditation.	Low
Demartini et al. (2015) (19)—A positive diagnosis of functional (psychogenic) tics	Retrospective case series; single center (Movement Disorders/Tourette clinic, National Hospital for Neurology and Neurosurgery, London, UK); 2011–2013		Case series of 11 adults (27.3% female) with functional tics at a quaternary movement disorders clinic, aiming to identify clinical features distinguishing functional from organic tics. All had abrupt adult-onset tics, no family/childhood tic history, and most had precipitating event (physical, psychological, or combined). Distinguishing features proposed: lack of premonitory urge, inability to suppress, absence of pali-/echo-/coprophenomena, “blocking tics” (>50%), atypical distribution, high rates of other FND symptoms (72.7%), and comorbid anxiety/depression. Neither anti-tic medication trial produced benefit.	High
Robinson & Hedderly (2016) (22)—Novel psychological Formulation and treatment of “tic attacks' in tourette syndrome	Case series; single center [Tic and Neurodevelopmental Movements Service (TANDeM), Evelina London Children's Hospital, UK]; 2014–2015		Case series describing “tic attacks” in 12 children with TS, proposing these episodes reflect a combination of tics and functional neurological movements triggered/maintained by anxiety and metacognitive processes rather than an extended bouts of typical tics. All had comorbid anxiety; management by hospital presentation and school avoidance was common. Detailed case report showed clinically meaningful improvement across tic severity, impairment, anxiety, depression, and QoL following metacognitive/CBT-based therapy targeting attention and threat interpretation rather than tics directly.	High
Ganos et al. (2016) (32)—“I swear it is Tourette's!”: On functional coprolalia and other tic-like vocalizations	Retrospective case series; single center (TS referral clinic, Hannover Medical School, Germany); 1995–2015		Retrospective case series of 13 patients (30.8% female, age at onset 5–50y) with prominent functional coprolalia and other complex tic-like vocalizations, all previously misdiagnosed with primary tic disorder (mostly TS). Distinguishing features included abrupt onset with physical/psychological precipitants, unusually varied coprolalic content, poor response to anti-tic medication, and a notable presence of premonitory sensations and symptom fluctuation. Cannabinoids showed unexpected benefit in 3 treatment-resistant cases.	Moderate
Okkels et al. (2023) (34)—Increased Number of Functional Tics Seen in Danish Adolescents during the COVID-19 Pandemic	Retrospective case series; single center (*N*ational Tourette Syndrome Clinic, Herlev University Hospital, Denmark); May 2020–June 2021	Yes —Copenhagen, Denmark	28 adolescents (96.4% female, mean onset age 14.4y) diagnosed with FTLB during the COVID-19 pandemic. Notable for unusually high rates of identifiable precipitating trauma (78.6%) and first-degree family psychiatric history (42.9%), alongside high social-media tic-content exposure (96.4%) and pandemic-related triggers (61.9% of those with a proximal trigger). Non-pharmacological treatment (psychoeducation + CBT elements) produced subjective improvement.	High
Andersen et al. (2023) (31)—Clarifying the Differences between Patients with Organic Tics and Functional Tic-Like Behaviors	Cross-sectional; single center (*N*ational Tourette Syndrome Clinic, Copenhagen University Hospital–Herlev, Denmark); May 2020–April 2022	Yes —Copenhagen, Denmark	53 FTLB patients vs. 200 TS patients [note: 23 of these 53 patients previously published in Okkels et al. (2023) (34)], examining differentiating features. Replicated established differences (older age at onset, female predominance, lower family tic history) and identified higher rates of family history of psychiatric diagnoses and higher rates of a psychosocial trigger (79.2% vs. 13.5%). After correcting for age and sex, psychiatric comorbidity rates did *not* differ significantly between groups.	High
Cavanna et al. (2022) (42)—Tourette Syndrome with Functional Overlay: A Case Series	Case series; single specialist center (Tourette Syndrome Clinic, National center for Mental Health, Birmingham, UK); March 2020–October 2022	Yes — Milan, Italy	10 patients (90% female) with primary TS who developed a functional tic overlay during the COVID-19 pandemic. Functional tics had significantly later onset (16.9 vs. 5.6y), acute/subacute onset (80% vs. 0%), absence of rostrocaudal distribution (20% vs. 100%), and more socially inappropriate behaviors (80% vs. 20%) compared to each patient's own neurodevelopmental tics. High rates of comorbid anxiety (70%), mood disorder (50%) and other FND symptoms (≥70%) were found.	High
Cavanna et al. (2023) (48)—New-­onset functional tics during the COVID-­ 19 pandemic: Clinical characteristics of 105 cases from a single center	Case series; single specialist center (Tourette Syndrome Clinic, National center for Mental Health, Birmingham, UK); April 2020–March 2023	Yes — Milan, Italy	105 patients developing functional tics during the COVID-19 pandemic. Confirms core FTLB phenotype: strong female predominance (72.4%), later/wider age range at onset (mean 21.4y, peak 12–26y), acute/subacute onset (75.2%), absence of rostrocaudal progression, high anxiety (69.5%) and mood disorder (40.0%) rates, frequent additional FND (41.0%), and substantial tic-related social media exposure (49.5%). ESSTS 2022 diagnostic criteria showed high sensitivity (84%) when retrospectively applied. A notable subgroup (22.9%) had comorbid pre-existing TS, and this subgroup differed from FTLB-only patients on family tic history (more likely), OCD/ADHD comorbidity (more likely), and several phenomenological features (less likely to have premonitory urges, suppressibility, intermittency).	High
Cavanna et al. (2023) (49)—Functional tics: Expanding the phenotypes of functional movement disorders?	Retrospective cross-sectional analysis; single specialist center (Tourette Syndrome Clinic, National center for Mental Health, Birmingham, UK); April 2020–April 2023	Yes — Milan, Italy	66 patients with COVID pandemic-onset functional tics vs. 44 patients with other functional movement disorders. FTLB group was significantly younger at onset (21 vs. 39y) with shorter illness duration and reported a nearly 50% social media tic-content exposure vs. 0%; comorbidity profiles were similar.	Moderate
Cavanna et al. (2023) (41)—Neurodevelopmental versus functional tics: A controlled study	Retrospective case-control study; single specialist clinic (Tourette Syndrome Clinic, National center for Mental Health, Birmingham, UK); April 2020–March 2023	Yes — Milan, Italy	83 patients with pandemic-onset functional tics vs. 83 age-/gender-matched TS patients. FTLB group showed higher anxiety, ASD, and comorbid FND rates, and lower family history of tic disorder, OCD/OCB, and ADHD. Absence of tic-related OCB and absence of family history of tics were the strongest predictors of FTLB diagnosis.	High
Cavanna et al. (2025) (11)—Neurodevelopmental Tics with Co-Morbid Functional Tic-like Behaviors: Diagnostic Challenges of a Complex Tourette Syndrome Phenotype	Retrospective case-control study of comorbid FTLB + TS; single specialist clinic (Tourette Syndrome Clinic, National center for Mental Health, Birmingham, UK); Jan 2023–Mar 2025	Yes — Milan, Italy	TS + FTLB patients vs. matched TS-only patients, examining ESSTS criteria applied to a comorbid “functional overlay” phenotype. FTLBs developing abruptly in patients with pre-existing TS on average 15 years after original tic onset. TS + FTLBs patients showed higher rates of complex vocal tics, coprolalia, self-injurious behavior, tic attacks, anxiety, ADHD, ASD, and other FND symptoms, while neurodevelopmental tic severity and family history of tics did not differ between groups.	High
Paulus et al. (2021) (24)—Pandemic Tic-like Behaviors Following Social Media Consumption	Case-control study; dual specialist center (University of Lübeck + TU Dresden, Germany); exact recruitment window not stated		13 patients with tic-like behaviors after social media consumption vs. 13 matched TS patients. Several phenomenological features highly discriminated FTLB from TS: abrupt onset, lack of symptom fluctuation over weeks/months, symptom worsening in the presence of others, and predominantly complex trunk/extremity movements. No group differences were found for premonitory sensations, suppressibility, complex vocalizations/coprolalia, comorbidities, or family history of tics.	Moderate
Fremer et al. (2022) (23)—Mass social media-induced illness presenting with Tourette-like behavior	Prospective case series; single specialist center (Tourette outpatient clinic, Hannover Medical School, Germany); May 2019–Sep 2021	Yes — Hannover, Germany; international registry	Patients framed by the authors as a “mass social media-induced illness” outbreak with YouTuber Jan Zimmermann proposed as a “virtual index case.” Unlike most FTLB cohorts, sex distribution was equal (50% female) — attributed to the male sex of the German index case. Nearly half (46.9%) had pre-existing TS. Psychiatric comorbidity was high (93.8%), and the study uniquely incorporated psychodynamic assessment.	High
Fremer et al. (2024) (20)—What distinguishes patients with mass social media-induced illness presenting with Tourette-like behavior from those with Tourette syndrome? Results of a prospective cohort study	Cross-sectional (prospective cohort vs. retrospective comparator group, single time point); single specialist center (TS outpatient clinic, Hannover Medical School, Germany); May 2019–September 2021	Yes — Hannover, Germany; international registry	Companion analysis paper to Fremer et al. 2022 (23), using the identical 32-patient FTLB cohort compared to a TS sample (*n* = 1,032). Compared to TS, the FTLB group showed significantly older age at onset (19.2 vs. 7.0y), higher female proportion (50% vs. 22.6%), higher rates of obscene/socially inappropriate symptoms (96.9% vs. 28.1%), and lower ADHD and OCD/OCB rates — but no significant differences in anxiety, depression, premonitory urges, or suppressibility.	Moderate
Hartung et al. (2026) (52)—Long-Term Follow-Up of Patients with Mass Social Media-Induced Illness Presenting with FTLB	Prospective longitudinal cohort study, single group; single center (TS outpatient clinic, Hannover Medical School, Germany); May 2019–March 2024	Yes — Hannover, Germany; international registry	Follow-up study of 30 of 53 social media induced-FTLB patients (mean interval 26.3 months, range 11–51) [cohort overlap with Fremer et al. 2022 (23)]. 77% reported improvement or complete remission, none reported worsening. Better outcomes were associated with younger age, male sex, shorter time-to-diagnosis, fewer comorbidities (especially absence of depression), and discontinuation of secondary gain. Neither receiving psychotherapy, psychotherapy type, pharmacotherapy, diagnostic acceptance, nor change in social media use was associated with outcomes.	High
Müller-Vahl et al. (2024) (12)—Functional Tic-Like Behaviors: A Common Comorbidity in Patients with Tourette Syndrome	Retrospective cross-sectional analysis; single specialist center (TS outpatient clinic, Hannover Medical School, Germany); 2002–2021	Yes — Hannover, Germany; international registry	71 patients with comorbid TS + FTLB, excluding social media-induced FTLB patients from Fremer et al. (2022) (23), compared to 1,032 TS-only patients. Distinct from typical de-novo/pandemic-era FTLB cohorts, FTLB emerged an 14.5 years after initial tics as a sudden, atypical symptom cluster. Nearly half were labeled “treatment-resistant TS” and 10% received DBS before FTLB was recognized. Compared TS-only patients, the TS + FTB group was more likely to be female, had more coprophenomena symptoms, more self-injurious behavior, and higher rates of comorbid OCD, with no significant difference in comorbidities.	Moderate
Hartung et al. (2024) (38)—Prevalence of mass social media-induced illness presenting with Tourette-like behavior	Cross-sectional population-representative self-report household survey in Germany; Aug–Dec 2021		Population-representative prevalence study of social media-induced FTLB (*N* = 9,253 surveyed across Germany, 2021). Prevalence 0.17% (strict criteria) to 0.36% (broad criteria). Notably male-predominant (76%–88% male), consistent with this center's hypothesis about the male “virtual index case” shaping German sex distribution differently than English-speaking countries. No participant had ever received a formal FND diagnosis.	Low
Buts et al. (2022) (37)—Paediatric tic-­ like presentations during the COVID-­ 19 pandemic	Retrospective case series; multi-site [Evelina London Children's Hospital, Great Ormond Street Hospital (UK) & Alberta Children's Hospital (Canada)]; 2020–2021	Yes — United Kingdom, multi-site; international registry	Case series describing the emergence of sudden-onset functional tic-like movements during the early COVID-19 pandemic. Found strong female predominance, late/adolescent onset, high tic severity, and low overall functioning at presentation. Near-universal psychiatric and/or neurodevelopmental comorbidity (91%), with prominent anxiety and ASD. High rates of pali/echo/copro-phenomena and a substantial subgroup with social-media-congruent movement patterns.	High
Larsh et al. (2022) (6)—Comparison of Impairment in Functional Tic Disorders Versus Tourette Syndrome	Retrospective cross-sectional case-control study; single center (pediatric movement disorders clinic, Cincinnati Children's Hospital, Ohio, USA); 2021	Yes—international registry	Youth with new-onset FTLB vs. age-/sex-matched TS patients. No differences were found on tic-related or non-tic-related impairment scores. However, FTLB group had higher rates of ED visits for movements (31% vs. 11%), homeschooling due to movements (14% vs. 2%), bodily injury from movements (49% vs. 17%), and current psychiatric/psychological treatment utilization.	High
Baizabal-Carvallo et al. (2023) (18)—Contrasting features between Tourette syndrome and secondary tic disorders	Retrospective, cross-sectional analysis of blinded video-recording; single center (Baylor College of Medicine, Houston, TX, USA); 3-year period (exact dates NR)		Video-based, blinded case-control study comparing 156 TS patients vs. 38 secondary tic disorder patients (21 with FTLB), excluding “TikTok tics” cases. TS patients were more male, younger at onset, had more complex/severe tics with greater cranial-cervical involvement and had higher rates of comorbid ADHD/OCD. Simple phonic tics had the best diagnostic accuracy for distinguishing TS from FTLB. Premonitory sensations were reported in only 19% of the FTLB subgroup.	High
Tomczak (2024) (25)—Time is ticking for TikTok tics: A retrospective follow-up study in the post-COVID-19 isolation era	Retrospective cohort study; single center (Boston Children's Hospital, MA, USA); 2020–2023		56 adolescents (96% female, 45% gender-diverse) with new-onset FTLBs beginning March 2020, with follow-up at a mean of 518 days. About ¾ showed improvement in tics and functioning at follow-up, largely independent of treatment modality (psychotherapy, CBIT, or SRI use all showed similar ∼76%–79% improvement rates), suggesting role of treatment-independent factors (e.g., reduced pandemic-era stress, return to in-person socialization). Gender-diverse patients showed comparable tic-specific improvement but poorer overall-functioning outcomes than cisgender patients. A subset of patients showed FTLB improvement but evolution into other functional neurological symptoms after tics resolved.	Moderate
Trau et al. (2022) (40)—Creating a Patient-Based Diagnostic Checklist for Functional Tics During the COVID-19 Pandemic	Retrospective cross-sectional analysis; single center (UNC Clinic for Tourette Syndrome and Tic Disorders, Chapel Hill, NC, USA); May 2020–Dec 2021		Study proposing and validating a novel diagnostic checklist to distinguish primary tic disorders (PTD, *n* = 119) from functional tic disorder (FTD, *n* = 36) (i.e., FTLB) and mixed tic disorder (MTD, *n* = 25) (i.e., TS + FTLB, or “functional overlay”). FTLB group was mostly female (94%), had later onset of tics, higher tic severity scores, lower rates of family history of tics, and higher self-reported anxiety/ADHD/OCB. No patient with both self-injurious behavior and coprophenomena met criteria for PTD; substantial minority of FTD patients reported no social media tic-content exposure.	High
Prato et al. (2023) (35)—Functional tic-like behaviours during the COVID-19 pandemic: Follow-up over 12 months	Prospective cohort; single center (outpatient Tourette clinic, Catania University, Italy); 2021–2022	Yes—international registry	11 patients with female-predominant, later-onset, abrupt FTLB presentation with high rates of echolalia/coprolalia/copropraxia. Mean IQ of 85.5. 12 nonth follow-up showed significant improvement in tic severity and anxiety symptoms, but not OCD or depressive symptoms.	Moderate
Owen (2022) (56)—Case report: Advice for schools…	Case series; single-center (TANDeM/Evelina London, UK); Jan 2019–Jan 2022	Yes — United Kingdom (multi-site); international registry	10 children/adolescents with FTLBs, presenting two detailed case reports and a clinician-developed school advice sheet emphasizing reduction of attention to tics and externalized attention strategies; 60% showed symptom reduction and all reported improved educational access after school intervention.	Moderate
Han et al. (2022) (5)—Rapid onset functional tic-like behaviours in children and adolescents during COVID-19…	Retrospective cross-sectional analysis; single center (Children's Hospital, Westmead, Sydney, Australia); Jan 2018–Jul 2021	Yes—international registry	22 FTLB patients vs. 163 TS patients, showing a sharp rise in FTLB prevalence from 2% (2018) to 36% (2021), observing female predominance, later onset, and significantly higher rates of anxiety, depression, self-injury, hospitalization, and school absenteeism.	Low
Mathew et al. (2023) (26)—Outcome in Pediatric Functional Tic Disorders Diagnosed During the COVID-19 Pandemic	Cross-sectional cohort with follow-up interviews; single center (University of Virginia, VA, USA); 2020–2021		29 pediatric FTLB patients with telephone follow-up. 48% had presented to the ED or urgent care for FTLB. Showed rapid but incomplete improvement after diagnosis (82% improved, median time-to-improvement 21 days), with older age and shorter time-to-diagnosis predicting faster improvement. Reported race/ethnicity of White (86%), Black (7%), Hispanic (3%), and “Other” (3%).	High
Firestone (2023) (4)—Rapid onset of functional tic-like behaviors among adolescent girls	Public health outbreak investigation (case series) (Minnesota high school, USA); onset Sept 2021, interviewed Oct–Nov 2021		CDC/Minnesota Dept. of Health outbreak investigation of a school-based cluster of 8 adolescent girls with rapid-onset FTLBs; near-universal history or current symptoms of anxiety/depression, no premonitory urges, and only modest prior social-media tic-content exposure (38%) — authors conclude echo phenomena via direct peer contact, not social media or recent COVID-19 infection, was the likely driver, and that underlying mental health vulnerability was central.	Moderate
Martino et al. (2023) (21)—The spectrum of functional tic-like behaviours: Data from an international registry	International multicenter retrospective registry; 10 tertiary referral centers, 8 countries (Canada, UK, Germany, Australia, USA, Italy, Hungary, France); Sep 2021–mid-2022	Yes—International registry includes Calgary, Canada; Hannover, Germany; UK multi-site; Sydney, Australia; Catania, Italy; Cincinnati, US	Multicenter international FTLB registry to date (*n* = 294), confirming rapid symptom escalation, high complex-movement/vocalization burden, marked female predominance (87%, except Hannover's 50%), high psychiatric/neurodevelopmental comorbidity and suicidality, and poor response to anti-tic pharmacotherapy; data support two FTLB subtypes — one linked to social media modelling (with more coprophenomena, complex phonic tics, premonitory urges, and remissions) and one unrelated to it.	High
Armstrong-Javors et al. (2024) (17)—Increase in Functional Tic Presentations in Sexual Orientation and Gender Identity Minority Youth…	Retrospective cross-sectional time series; single-center (MGH Movement Disorder Clinics, Boston, USA); May 2018–Jan 2022		Showed an 8.6-fold rise in new functional tic presentations from pre- to post-pandemic (2 to 17 cases) while developmental tic presentations remained stable (114 vs. 112); 37% of the FTLB cohort identified as a sexual orientation/gender identity minority (versus an estimated 3%–15% population base rate).	Moderate
Pringsheim & Martino (July 2021) (39)—Rapid onset of functional tic-­ like behaviours in young adults during the COVID-­ 19 pandemic	Cross-sectional comparative study with prospective enrollment; single center (University of Calgary Tic Registry, Canada); 2021	Yes — Calgary, Canada; international registry	9 young adult females with rapid-onset FTLB vs. 24 adults with TS; FTLB group showed significantly worse tic severity, impairment, and quality-of-life scores, higher self-reported ADHD/anxiety/OCD symptom burden (though not with corresponding diagnoses except depression), and universal social media tic-content exposure.	High
Pringsheim et al. (Aug 2021) (3)—Rapid Onset Functional Tic-Like Behaviors in Young Females During the COVID-19 Pandemic	Cross-sectional analysis of prospective registry data; (University of Calgary Tic Registry, Canada); 2012–2021	Yes — Calgary, Canada; international registry	Viewpoint drawing on Calgary registry data (20 FTLB vs. 270 TS); FTLB group showed later onset, female predominance, higher tic severity/impairment, and higher anxiety/depression; reports 100% social media exposure.	High
Nilles et al. (2024) (53)—Prospective follow-­ up study of youth and adults with onset of functional tic-­ like behaviours during the COVID-­ 19 pandemic	Prospective cohort follow up (University of Calgary Tic Registry, Canada); Oct 2020–Dec 2022, followed to Apr 2023	Yes — Calgary, Canada; international registry	12-month follow-up of 83 youth/adults with FTLBs, showing significant improvement in tic severity and repertoire contraction from baseline, though 19%–25% worsened at each 6-month interval. Younger age at onset (<18y) and higher baseline tic severity were the strongest predictors of improved 6-month outcome; presence of other functional neurological symptoms predicted worse outcome.	High
Nilles et al. (2024) (43)—What are the Key Phenomenological Clues to Diagnose Functional Tic-Like Behaviors in the Pandemic Era?	Cross-sectional analysis of prospective registry data; (University of Calgary Tic Registry, Canada); Oct 2020–Dec 2022	Yes — Calgary, Canada; international registry	Phenomenology study comparing tic inventories of 41 FTLB vs. 195 TS patients; identified copropraxia, saying words, coprolalia, popping noises, whistling, simple head movements, and self-injurious behaviors as most strongly associated with FTLB diagnosis, with simple head movements and word enunciation remaining independently associated in the age-adjusted multivariable model; throat clearing was more suggestive of TS.	High
Szejko et al. (2024) (50)—Premonitory Urge in Patients with Tics and Functional Tic-Like Behaviors	Cross-sectional comparison within prospective cohort; (University of Calgary Tic Registry, Canada); 2017–2023	Yes — Calgary, Canada; international registry	Compared self-reported premonitory urges between 26 FTLB-only patients vs. 14. FTLB + TS patients vs. 83 TS patients; found no significant difference in premonitory urges across all three groups.	High
Berg et al. (2024) (36)—Psychological Factors Associated with Functional Tic-like Behaviours during the COVID-19 Pandemic	Cross-sectional analysis; (University of Calgary Tic Registry, Canada); Oct 2020–Jun 2022	Yes — Calgary, Canada; international registry	FTLB, TS, and neurotypical control groups compared by psychological/psychiatric factors; found markedly higher rates of depression, panic disorder, borderline personality disorder, and insecure attachment in FTLB vs. control; no significant group differences in coping, suggestibility, or social media use/exposure to tic content; notable representation of gender diverse identity in the FTLB group (42.8%), with no significant psychiatric differences between cisgender and gender diverse subgroups.	High
Ducroizet et al. (2025) (51)—Outcomes of functional tics in adolescents: a single-center tertiary study	Longitudinal follow-up study (quality-improvement design, no comparator group); single-center (TANDeM/Evelina London Children's Hospital, UK), diagnosed Jan 2020–Mar 2023	Yes — United Kingdom (multi-site); international registry	43 patients with follow-up. Psychiatric comorbidity rose over time (77% at follow-up) with more anxiety disorders (70%) rather than depression (12%). Notable co-occurrence with other FND symptoms (75% at follow-up, up from 60%), consistent with “phenoconversion.” Prognosis was mixed: ∼65% improvement/recovered, but a significant minority had poor outcomes (16% no change, ∼19%–21% worsening); overall functioning improved, but the average patient remained in the mild-moderate impairment range. Functional impact on school attendance and parental employment persisted at follow-up.	Low
Manbeck & Samanta (2025) (57)—Functional Tic-Like Behaviors in Youth With Gender Dysphoria: A Case Series	Retrospective case series; single center (Tourette Centers of Excellence, Arkansas Children's Hospital, USA); Jan 2022–Dec 2023		9 patients with FTLB onset closely tracking onset of gender dysphoria diagnosis/identity milestones (median 4 months, all within 6 months). High psychiatric comorbidity (100% anxiety and depression), with notably high suicidality (44% ideation, 33% attempts). Outcomes were favorable with multidisciplinary care (100% resolution/near-resolution by 12 months).	High
Duncan et al. (2024) (63)—Evaluation of a psychoeducation group for children presenting with functional tic-like behaviours	Pre-post intervention, uncontrolled, single-arm pre-post; single center (Great Ormond Street Hospital, UK; May 2021–Jun 2022	Yes — United Kingdom (multi-site); international registry	Uncontrolled service evaluation of a one-off remote psychoeducation intervention for pediatric FTLB patients, families, and professionals at a UK specialist tic service. Primary outcome was goal-based outcomes, which showed a large pre-post improvement.	Moderate
Maxwell et al. (2023) (64)—Integrated cognitive behavioral intervention for functional tics (I-CBiT): case reports and treatment formulation	Pre-post intervention, uncontrolled; single center (UNSW-affiliated specialist tic/FND outpatient clinic, Australia); 2019–2023		8 patients received a novel integrated CBT intervention (I-CBiT) combining traditional tic-behavioral therapy with CBT-based urge acceptance. All showed reductions in tic severity and impairment, with improved suppressibility, function and school return. New functional symptoms emerged as tics resolved in 3 cases, with one case of emergent suicidality coinciding with tic improvement.	Moderate

**Table 2:** Demographics, history, and clinical course.

	Demographics			Psychiatric and Neurologic History		Characteristics, Influences, and Outcomes		
Authors (Year)—Paper	N	Mean Age of FTLB Onset ± SD (Range)	% Female/Gender	Comorbidity	Tic History	Clinical Course	% Exposed to Online Tic Content	Treatment
Mejia & Jankovic (2005) (33)—Secondary tics and tourettism	16	NR by subgroup	NR by subgroup	Psychogenic dystonia (6.25%)	NR by subgroup	NR by subgroup	N/A	NR
Demartini et al. (2015) (19)—A positive diagnosis of functional (psychogenic) tics	11	37.2 ± 13.5 (16–65)	27%	Any psychiatric comorbidity (72.7%); Depression (45.5%); Anxiety (27.3%); OCD (0%); ADHD (0%); Other FND symptoms (72.7%)	Prior personal history of childhood tics (0%); Family history of TS or other tic disorder (0%)	NR	NR	Prior anti-tic medication trials with no benefit (18.2%)
Robinson & Hedderly (2016) (22)—Novel psychological Formulation and treatment of “tic attacks' in tourette syndrome	12	11.25 ± 2.3 (7–15)	25%	Any anxiety (100%); Social anxiety disorder (50%); OCD (33.3%); Depression (16.7%); Headaches (16.7%); Specific phobias (8.3%); ASD (8.3%)	Comorbid TS (100% by inclusion criterion)	Tic attack frequency: occasional (50%), weekly (25%), daily (25%) Previous management included ED presentation (41.7%), school avoidance (58.3%)	NR	*N* = 1 received therapy
Ganos et al. (2016) (32)—“I swear it is Tourette's!’: On functional coprolalia and other tic-like vocalizations	13	25.31 ± 14.34 (5–50) [calculated]	31%	Other FND symptoms at follow-up (38.5%); ADHD (30.8%); OCD/OCB (7.7%)	Comorbid TS (100%); No family history of tic disorders (0%)	Symptom fluctuations including instances of full remission during holidays with abrupt recurrence upon returning home (84.6%)	NR	Anti-tic medication trialed with no improvement, or atypical adverse effects (76.9%); Cannabinoids with marked improvement (23%)
Okkels et al. (2023) (34)—Increased Number of Functional Tics Seen in Danish Adolescents during the COVID-19 Pandemic	28	14.4 (10.9–18.3)	96%	Any psychiatric symptoms/diagnoses (78.6%); OCD (28.6%); ADHD (25.0%); Anxiety (21.4%); Depression (21.4%) [calculated]	Prior personal history of childhood tics (50%); Family history of tics (10.7%)	Trauma/precipitating event ≥1 year prior to onset (78.6%; e.g., bereavement, bullying, family conflict); had separate proximal triggers (95.2%; 61.9% were pandemic-related). Severity reduction or complete resolution at follow-up (100%)	96.4%	Psychoeducation + CBT elements; Encouraged to stop following tic-related social media; Referred to mental health services (43%); No pharmacological treatment used.
Andersen et al. (2023) (31)—Clarifying the Differences between Patients with Organic Tics and Functional Tic-Like Behaviors	53	13.7 ± 2.4 (6.0–19.8)	94%	ADHD (18.9%); Anxiety (26.4%); ASD (13.2%); OCD (18.9%); Other psychiatric diagnoses (28.3%)	Personal history of tics (0% by exclusion criteria); Family history of tics (11.3%)	Psychosocial trigger immediately before/at onset (79.2%) [OR = 59.62 compared to TS group] E.g.: pandemic, exams, bullying, family conflict.	56.6%	NR
Cavanna et al. (2022) (42)—Tourette Syndrome with Functional Overlay: A Case Series	10	16.9 ± 2.7 (12.0–22.0)	90%	OCD (30%); OCB (80%); ADHD (30%); ASD (20%); Mood disorder (50%); Anxiety disorder (70%); Other FND (30–70%)	Comorbid TS (100% by inclusion criterion); Family history of tics (40%)	NR	NR	Pharmacotherapy (40%): SSRIs, anti-DA, alpha-2 agonist, beta-blocker
Cavanna et al. (2023) (48)—New-­onset functional tics during the COVID-­ 19 pandemic: Clinical characteristics of 105 cases from a single center	105	21.4 ± 10.8 (11.0–61.0)	72%	Anxiety disorder (69.5%); Mood disorder (40.0%); ASD (26.7%); ADHD (18.1%); OCD (9.5%); OCB (22.9%) Other FND (41.0%)	Comorbid TS (22.9%); Family history of tic disorder (14.3%)	Acute/subacute onset + peak severity within 1 month (75.2%); Psychological trigger at onset (72.4%); Tic attacks (32.4%)	50%	Pharmacotherapy (55.2%): SRIs (36%), anti-DA (21%), alpha-2 agonists (7%), pregabalin (5%); Psychotherapy (39.0%): CBT-based, targeting anxiety/mood.
Cavanna et al. (2023) (49)—Functional tics: Expanding the phenotypes of functional movement disorders?	66	21.1 ± 10.6 (11–61)	71%	Anxiety disorder (69.7%); Mood disorder (30.3%); Functional seizure disorder (33.3%)	Personal history of tics (0% by exclusion criteria); Family history of movement disorders (7.6%)	NR	47%	Pharmacotherapy (48.5%): mostly SRIs; Psychotherapy (37.9%)
Cavanna et al. (2023) (41)—Neurodevelopmental versus functional tics: A controlled study	83	21.2 ± 10.9 (11–61)	71%	Any psychiatric comorbidity (90.4%); Anxiety disorder (69.9%); Mood disorder (38.6%); Other FND (47%); ASD (25.3%); ADHD (10.8%); OCB (7.2%); OCD (3.6%)	Personal history of tics (0% by exclusion criteria); Family history of tic disorder (7.2%)	NR	NR	Pharmacotherapy (56.6%); Psychotherapy (38.6%)
Cavanna et al. (2025) (11)—Neurodevelopmental Tics with Co-Morbid Functional Tic-like Behaviors: Diagnostic Challenges of a Complex Tourette Syndrome Phenotype	63	22.4 ± 9.4	70%	OCD (20.6%); OCB (73.0%); ADHD (46.0%); ASD (30.2%); Mood disorder (52.4%); Anxiety disorder (71.4%); Other FND (22.2–30%)	Comorbid TS (100% by inclusion criteria); Family history of neurodevelopmental tics (50.8%)	NR	NR	Psychotherapy (31.7%); Pharmacotherapy (65.1%): included: SRIs (30.2%), anti-DA (20.6%) alpha-2 agonists (9.5%)
Paulus et al. (2021) (24)—Pandemic Tic-like Behaviors Following Social Media Consumption	13	15.31 ± 2.96	38.5%	ADHD (7.7%); OCD (23.1%); ASD (7.7%)	Comorbid TS (0% by exclusion criteria); Family history of tics (7.7%)	NR	100% by inclusion criterion	Anti-DA medication (46.2%); of those treated, symptom improvement in 33.3%
Fremer et al. (2022) (23)—Mass social media-induced illness presenting with Tourette-like behavior	32	19.2 ± 11.0 (10–53)	50% (2 patients — 1 male, 1 female — self-identified as non-binary; assigned to biological sex for analysis)	Any psychiatric symptom (93.8%); Severe mental illness (40%); Abnormalities in social behavior (81.3%); OCB/OCD (46.9%); Anxiety (40.6%); Depression (31.3%); ASD (15.6%); Personality disorder (18.8%); Suicidal ideation (15.6%); Intellectual disability (9.4%); PTSD (6.3%)	Comorbid TS (46.9%)	At follow-up (mean 4.8mo): improvement in 53.1%; average improvement was 74.3%. Older age + more comorbidities had worse prognosis. 12.5% improved immediately after receiving diagnosis; 40.6% improved with treatment; 78.1% accepted diagnosis and 15.6% insisted on TS diagnosis; 46.9% reported accidental self-injury.	100% by inclusion criterion. 90.6% reported watching the “Gewitter im Kopf” YouTube channel prior to symptom onset	Anti-tic medication prior to correct FND diagnosis (62.5%); Psychotherapy (28.1%); Other unspecified treatment (12.5%)
Fremer et al. (2023) (23)—What distinguishes patients with mass social media-induced illness presenting with Tourette-like behavior from those with Tourette syndrome? Results of a prospective cohort study	32 [same sample as Fremer et al. 2022 (23)]	19.2 ± 11.01 (10–53)	50%	ADHD (9.0%); OCB/OCD (46.9%); Anxiety (40.6%); Depression (31.3%)	Comorbid TS (46.9%)	NR	100% by inclusion criterion	NR
Hartung et al. (2026) (52)—Long-Term Follow-Up of Patients with Mass Social Media-Induced Illness Presenting with Functional Tic-like Behaviors	30 [from a baseline pool of *N* = 53 patients seen 2019–2023; of these, 32 were previously published Fremer et al. 2022 (23)]]	NR for age of onset; age at follow up: 22.5 (14–57)	60.0% (gender identity includes 10.0% non-binary, 3.3% transgender man)	Other FND (43.3%); Depression (53.3%); Anxiety (53.3%); OCD/OCB (33.3%); PTSD (26.7%); ADHD (20.0%); ASD (20.0%); Personality disorder (13.3%); Eating disorder (10.0%); Psychosis (3.3%)	Comorbid TS (36.7%)	At follow up (mean 26.3mo): complete remission (23.3%); much improved (53.3%); minimally improved (10.0%); no change/same as baseline (13.3%); worse (0%). 68% felt subjectively improved. Most showed slow/gradual improvement (56.7%), some had sudden improvement (23.3%). Males showed greater improvement than females; no difference by gender identity. Younger age associated with better outcome.	100% by inclusion criteria. Change in social media use did not show a significant association with outcome.	Psychotherapy (86.7%): outpatient (80.0%), inpatient (30.0%), other therapy (30%), tic-oriented interventions (23.3%). No difference in improvement between therapy vs. no therapy. No difference between FTLB-specific vs. nonspecific therapeutic focus. Pharmacotherapy at baseline (63.3%); at FU ranged from fully discontinued (20%) to added new medication (20%); Among *n* = 13 on medication at FU, 84.6% reported no effect on FTLB specifically; self-medicating with cannabis reported FTLB improvement (15.4%)
Müller-Vahl et al. (2023) (12)—Functional Tic-Like Behaviors: A Common Comorbidity in Patients with Tourette Syndrome	71 (excludes sample from Fremer et al. 2022, 2023) (23)]	20.8 ± 11.8 (5–52)	38%	OCB (58%); Other FND symptoms (34%); ADHD (37%); Depression (32%); Anxiety (27%); OCD (24%); ASD (3%); Unconscious intrapsychic conflicts (79%); emotional dysregulation (56%).	Comorbid TS (100% by inclusion criteria); Tic-like behaviors included self-injurious behavior (55%)	Onset described as sudden worsening after long stable disease course (86%); Precise date of onset identifiable (54%); Ability to differentiate tics from FTLB after diagnosis (48%); Complete cessation of FTLB in specific situations (62%); Symptoms markedly improved during clinical exam (72%)	0% by exclusion criteria	Received DBS for presumed treatment-resistant tics (10%); Mean number of different anti-tic pharmacological agents trialed: 4.9; Atypical/unusual side effects with anti-tic medication (28%)
Hartung et al. (2024) (38)—Prevalence of mass social media-induced illness presenting with Tourette-like behavior in Germany between 2019 and 2021	33 (“probable” of *N* = 9,253 surveyed); Nested subset of 16 (“most likely”)	Probable: 30.5 ± 14.6 (10–58); Most likely: 25.6 ± 11.5 (11–46)	Probable: 24.2% Most likely: 12.5%	Other FND (0%)	NR	Complete remission (6.3–12.1%); improvement (0–12.1%); no change (18.8–27.3%); waxing/waning (21.2–37.5%), deterioration (12.5–15.2%) [calculated]	100% by inclusion criteria	Any treatment initiated (19–33%)
Buts et al. (2022) (37)—Paediatric tic-­ like presentations during the COVID-­ 19 pandemic	34	13.7 ± 2	94%	Any neuropsychiatric comorbidity (91.0%); Anxiety (50%); Depression (24%); ASD (12–57% per site); ADHD (57% in one site)	Prior personal history of tics (44.0%); Family history of tics (29.0%)	NR	77%	NR
Larsh et al. (2022) (6)—Comparison of Impairment in Functional Tic Disorders Versus Tourette Syndrome	89	NR; age at assessment: 15.6 ± 2.0	93.3%	(via parental report) ADHD-associated behavior (50.0%); Obsessive behavior or anxiety (74.4%); Compulsive behaviors (29.8%)	NR	Emergency department presentation (31%); self-injurious behaviors (49%). Both significantly higher than TS group.	NR	Psychiatric treatment (41.9%); psychological treatment (62.1%). Both higher than in TS group.
Baizabal-Carvallo et al. (2023) (18)—Contrasting features between Tourette syndrome and secondary tic disorders	21	31.62 ± 15.25	47.7%	ADHD (14.3%); OCD (23.8%)	Comorbid TS (0%) by exclusion criteria	NR	0% by exclusion criteria	NR
Tomczak (2024) (25)—Time is ticking for TikTok tics: A retrospective follow-up study in the post-COVID-19 isolation era	56	14 ± 1.9	96% (45% identified as gender-diverse: nonbinary or transgender, mostly trans-male)	Anxiety (93%); Depression (71%); ADHD (45%); ASD (7%); OCD (23%); Functional seizures (32%); Other FND symptoms (7- 23%)	Personal history of tics (32%)	Much improved at follow-up (79%); significantly impaired at follow-up (29%). Gender-diverse patients had less improvement in function. Major life stressor reported prior to onset (54%)	NR	Psychotherapy (86%): CBIT in (34%), no additional benefit found for CBIT over nonspecific therapy.; Pharmacotherapy (91%): improvement on SRI (76%); improvement from therapy without SRI (78%) — no difference in improvement by treatment modality or combination.
Trau et al. (2022) (40)—Creating a Patient-Based Diagnostic Checklist for Functional Tics During the COVID-19 Pandemic	61 (36 FTLB only; 25 FTLB + TS)	14 ± 2	94%	(Via patient/family report) FTLB Only: Anxiety (89%); ADHD symptoms (69%); OCB (56%); other FND (17%) Comorbid TS & FTLB**:** Anxiety (100%); OCB (96%); ADHD symptoms (68%); other FND (16%)	Comorbid TS (41.0%); Family history of tics (19%)	≥25% improvement at follow-up (29%)	62% reported exposure to videos of tics on social media prior to tic onset, 12% after tic onset, and 25% had no exposure (of *n* = 16 who provided data).	Anti-tic pharmacotherapy showed poor response relative to TS group
Prato et al. (2023) (35)—Functional tic-like behaviours during the COVID-19 pandemic: Follow-up over 12 months	11	14.0 ± 2.6	73%	Comorbid FND (63.6%); anxiety/depression/OCD NR categorically	Prior personal history of childhood tics (45.4%); Family history of tic disorder (36.4%)	≥25% reduction in tic severity (45.5%)	36.4%	CBT (100% during follow up). Pharmacotherapy (73% prior to study, 18% during follow up) with reported improvement in anxiety, sleep, partial tic relief. Anti-tic medication generally described as ineffective.
Owen (2022) (56)—Case report: Advice for schools on managing functional tic-like behaviours	10	12.3 ± 1.8 (9–14) [calculated]	100%	Other FND symptoms (80%); Anxiety (70%); ADHD (20%); Depression (10%); ASD (10%); OCD (10%); Self-harm (20%)	Comorbid TS (30%)	Symptom reduction (60%); reported improved educational access following school-based intervention (100%)	NR	School-based accommodations (100%); psychotherapy (50%); anti-tic pharmacotherapy (0%)
Han et al. (2022) (5)—Rapid onset functional tic-like behaviours in children and adolescents during COVID-19: Clinical features, assessment and biopsychosocial treatment approach	22	13.8	100%	Anxiety/depression (95.0%); OCD (22.7%); ADHD (13.6%); ASD (9.1%); Suicidal ideation (31.8%); Panic attacks (22.7%); hallucinations (13.6%)	Comorbid of TS (27.0%); Family history of TS (13.6%)	Self-injurious behaviors (50 vs. 4%); hospitalization/ED presentation (36% vs. 2%); school absenteeism (55% vs. 7%) compared to TS group. At follow-up (2mo–3 yr): persistent symptoms (68.2%); partial improvement (18.2%); complete resolution (13.6%)	18.2%	1 medication trial (18.2%), 2 medications (27.2%), ≥3 medications (31.8%); 27.2% referred to specialized FND rehabilitation program.
Mathew et al. (2023) (26)—Outcome in Pediatric Functional Tic Disorders Diagnosed During the COVID-19 Pandemic	29	NR; age at diagnosis: 15.8 ± 1.4	89.7% (gender diversity in 10%)	Mood disorder (69%); Anxiety disorder (66%); Other functional symptoms (34.5%); ADHD (10.3%)	Family history of tics (13.0%)	ED or urgent care presentation (48%); Some improvement at follow-up (82.0%); median time to initial improvement was 21 days post-diagnosis. Older age at diagnosis and shorter time-to-diagnosis associated with improvement.	55.2%	Pre-diagnosis: many with anti-tic medication + psychotherapy; Post-diagnosis: CBIT (17.2%)
Firestone (2023) (4)—Rapid onset of functional tic-like behaviors among adolescent girls—Minnesota, September–November 2021	8	Median 16 (15–17)	100%	Anxiety diagnosis (63%) Depression diagnosis (50%); ADHD (25%); OCD (13%)	Comorbid TS (0%)	Interview was median 7 days after symptom onset (range 3–24); 1/8 (12.5%) resolved by time of interview.	63%	All referred to mental health professionals for evaluation; FTLB educational webinar offered to parents/school.
Martino et al. (2023) (21)—The spectrum of functional tic-like behaviours: Data from an international registry	294	NR; age at assessment: 15.1 ± 5, median 14 (8–53)	87%	Any psychiatric comorbidity (80%); Anxiety Disorder (66%); Other FND (32%); Mood Disorder (28%); Suicidal ideation (27%); suicidal attempt (10%); ASD (24%); ADHD (23%); OCD (9%);	Comorbid TS (19%); Family history of TS (12%)	Peak severity reached within 1 month (70.0%); median symptom duration of 12mo (range 1–192mo); spontaneous remission occurred in 20.0%, of whom 61.7% had recurrence.	57%	Anti-tic pharmacotherapy (41%)
Armstrong-Javors et al. (2024) (17)—Increase in Functional Tic Presentations in Sexual Orientation and Gender Identity Minority Youth During Coronavirus Disease 2019	19	14.25 (10–26)	95% (10% transgender)	ADHD (47%); OCD (42%); Other FNDs (37%)	Comorbid TS (21.0%)	NR	NR	NR
Pringsheim & Martino (July 2021) (39)—Rapid onset of functional tic-­ like behaviours in young adults during the COVID-­ 19 pandemic	9	15.3, 10.7–20 (95% CI)	100%	Overall psychiatric comorbidity (89%); ADHD diagnosis (22.0%); OCD diagnosis (0%); anxiety diagnosis (56.0%); depression diagnosis (44.0%); substance use disorder (11.0%); no psychiatric comorbidity (11.0%)	Prior personal history of childhood tics (22.0%)	Acute onset (100%); suggestibility and distractibility (100%); tic severity, functional impairment, and quality of life all worse than TS group.	100%	CBIT-based approach adapted; psychoeducation for patient/family; treatment of comorbid anxiety/mood where present.
Pringsheim et al. (Aug 2021) (3)—Rapid Onset Functional Tic-Like Behaviors in Young Females During the COVID-19 Pandemic	20	13.9, 13.1–14.7 (95% CI)	95%	Anxiety (75%); Depression (55%); ADHD (25%); OCD (5%); ASD (0%)	Prior personal history of childhood tics (15%)	Tic severity and impairment significantly higher than TS group.	100%	*α*-agonist (35.0%); anti-DA (15.0%); SRI (45.0%, higher than TS group); stimulant (15.0%).
Nilles et al. (2024) (53)—Prospective follow-­ up study of youth and adults with onset of functional tic-­ like behaviours during the COVID-­ 19 pandemic	83	NR; age at first visit: 18.0 ± 5.6 (11–­ 53); adolescents *n* = 41, adults *n* = 42	96% (23% identified as trans or gender diverse.	Anxiety disorder (65%); Depressive disorder (42%); Other FND (30%); ADHD (35%); OCD (7%); ASD (4%)	Prior personal history of childhood tics (27%)	Overall improvement in tic severity at 6 and 12mo; predictors were age of onset <18y, CBT for anxiety/depression. 19–25% worsened; predictors were higher baseline severity, other FND symptoms.	100%	SRIs (47.0–61.0%); alpha-agonists (10–22%); anti-DA (13.0–20.0%);stimulants (15–16%); CBT for anxiety/depression (67.0%), CBIT (13%)
Nilles et al. (2024) (43)—What are the Key Phenomenological Clues to Diagnose Functional Tic-Like Behaviors in the Pandemic Era?	41	16.1 (11–20)	98%	NR	Prior history of tics in some patients (not quantified)	NR	NR	NR
Szejko et al. (2023) (50)—Premonitory Urge in Patients with Tics and Functional Tic-Like Behaviors	40	16.8 ± 5.7	90% (17.5% identified as gender diverse)	NR	Comorbid TS (35%)	No difference in premonitory urge between groups; Total tic severity was higher in FTLB than TS group.	NR	NR
Berg et al. (2024) (36)—Psychological Factors Associated with Functional Tic-like Behaviours during the COVID-19 Pandemic	35	NR; age at assessment: 17 ± 3.1	91.4% (42.8% identified as gender diverse)	MDD (53–72%); Borderline Personality Disorder (45.5%); GAD (37.5%); Bipolar I Disorder (12.5%); Panic Disorder (65.6%); Agoraphobia (43.8%); Social Anxiety Disorder (37.5%); OCD (21.9%); PTSD (18.8%); Alcohol/Substance Use Disorder (>15.6%); Other FND symptoms (8.6%); Eating disorder (>6.3%)	Family history of TS (12.1%)	NR	NR	Pharmacotherapy (74%): SRIs (51.4%); other antidepressants (17.1%); anti-DA (17.1%); alpha-agonists (8.6%)
Ducroizet et al. (2025) (51)—Outcomes of functional tics in adolescents: a single-center tertiary study	43	13 ± 1.7	100% (7% identified as gender diverse)	≥1 psychiatric comorbidity (77% at follow-up, up from initial 60%); Other FND symptoms (75% at follow-up, up from 60%); GAD (67%); OCD (12%); Depression (12%); Panic disorder (9%); ASD (19%); ADHD (9%)	NR	Improved or fully resolved (∼2/3); complete resolution (14%), stayed the same or got worse (∼1/3). Many had lasting issues with school attendance and parental work. Other FNDs often increased in frequency by follow-up.	NR	Open to mental health services at follow-up (51%); Informed schools implemented adaptations (95%)
Manbeck & Samanta (2025) (57)—Functional Tic-Like Behaviors in Youth With Gender Dysphoria: A Case Series	9	NR; age of presentation: 14.2 (9–18)	88.9% (100% gender dysphoria by inclusion criteria)	Anxiety (100%); Depression (100%); ADHD (44.4%); Suicidal ideation (44.4%); Prior suicide attempt (33.3%); PTSD (22.2%); OCD (22.2%); Eating disorder (11.1%); ASD (11.1%)	Comorbid TS (11%)	Complete or near-complete resolution at ≥12mo follow-up (100%); Mental health stabilized/improved (88.9%)	NR	Psychotherapy (100%): complete resolution with therapy alone (22%). Pharmacotherapy (78%), most commonly SRIs and alpha-2 agonists for anxiety, depression, ADHD rather than FTLBs directly. History of prior trialed/discontinued medication (56%)
Duncan et al. (2024) (63)—Evaluation of a psychoeducation group for children presenting with functional tic-like behaviours	58	NR; age at assessment: 14.3 ± 2	85.0% (10.5% identified as gender diverse)	NR	NR	NR	NR	One-off 2.5-hour remote group psychoeducation + CBT-informed intervention. Parents found helpful (80.9%), children understood what functional tics are post-group (100%) vs. adults (85%).
Maxwell et al. (2023) (64)—Integrated cognitive behavioral intervention for functional tics (I-CBiT): case reports and treatment formulation	8	14.6 ± 2.39 [calculated]	100% (12.5% identified as gender diverse)	GAD (75%); Social anxiety (62.5%); Panic symptoms (62.5%); Other functional symptoms (50%); MDD (37.5%); OCBs/OCD (37.5%); ASD (25%); Self-harm behaviors (12.5%); Selective mutism history (12.5%)	Prior personal history of childhood tics (25%)	Total tic severity, frequency, and functional impairment significantly improved. Tic-free or near tic-free post-treatment (50%). Symptom replacement occurred (38%); *n* = 1 developed new suicidal ideation as tics improved.	37.5% (*n* = 1, tics became part of online identity)	Novel 3-phase intervention combining behavioral tic therapy with CBT targeting stress-arousal system; total sessions ranged 9–18 per case; duration 2–8 months; adjunct pharmacotherapy in 2/8 cases.

### Demographic factors

While the tics of TS predominantly emerge in childhood, the extant literature suggests that FTLBs primarily begin in adolescence with an average age between 11 and 37 years, and most sample means clustering between 13 and 15 years of age (range: 5 years—65 years) (17–22). Additionally, FTLBs were observed to affect girls more than boys, with clinical registries suggesting that biological sex ratios vary between 7:1 and 9:1 females to males (with some exceptions among smaller samples, especially when rapid onset FTLBs were excluded) (18, 21, 23, 24). Alongside biological sex, several reports found that FTLBs are more present among gender diverse individuals with rates varying across studies (37% to 45%) (17, 25). Interestingly, when compared to patients with TS, one report identified that gender diversity was more strongly associated with FTLBs compared to biological sex (17). Given that FTLBs were identified internationally, race and ethnicity status of individuals affected by FTLBs was not consistently collected and/or reported. One study did observe that individuals across race and ethnicities are affected by FTLBs (e.g., 86% white, 7% Black, 3% Hispanic, and 3% “other”) (26).

### Psychosocial factors

While TS is associated with genetic and neurobiological factors (27, 28)**,** a variety of psychosocial factors can influence the expression of both TS and FTLB symptoms (29, 30) and individuals with FTLBs may have a more pronounced sensitivity. For instance, internal factors such as stress and anxiety have been strongly linked to increased symptom expression (21, 22, 25, 31–35). Indeed, up to 80% of individuals identified specific psychosocial precipitants to FTLB onset (e.g., worries about school, family conflict, bullying) (21, 25, 31, 34, 35). Additionally, individuals with FTLBs were observed to have frequent insecure attachment styles (36), intense affective reactivity (36), and autonomy-dependency conflicts (12, 23).

A strong association emerged between social media use and onset of FTLBs (4, 21, 23, 24, 31, 34, 37–40). Indeed, the largest international registry reported that over 57% of patients with FTLBs viewed tic-related social media content prior to FTLB onset (21). Many patients exhibited the same complex phonic tics as social media influencers (38, 39), with case-control comparisons suggesting that FTLBs are influenced by social media and other social factors (24).

Beyond social learning via social media, other socio-contextual factors have been suggested to contribute to FTLBs. For instance, one report from a large TS clinic identified an 8.6-fold increase in the incidence of FTLBs starting in 2020 with the COVID-19 pandemic, as compared to their pre-pandemic population (17). Another report identified that 62% of patients with FTLBs reported that the COVID-19 pandemic and its related social isolation sequelae were associated with FTLB symptom onset (34).

### Phenomenology and clinical course

Individuals with TS and those without TS can both exhibit FTLBs (11, 12, 41), and many patients exhibited a mixed presentation of both typical tics and FTLBs. However, evidence suggests that the phenomenology and clinical course of FTLBs is notably different from the tics of TS. FTLBs primarily have an abrupt onset that consists of complex motor movements and vocalizations (21, 39, 42). However, FTLB presentations span the full range from simple motor tics (face, limb, and trunk movements) and simple phonic tics (whistling, sniffing, and animal sounds) to strikingly complex presentations—directed actions including hitting, throwing, kicking, and self-injurious behaviors like head-banging and self-biting, alongside elaborate vocalizations including context-bound and socially directed phrases, coprolalia, and echolalia/palilalia (21, 23). Notably, self-injurious behaviors, coprophenomena, tic attacks, lack of rostro-caudal progression, context-dependent and socially salient tics are strongly associated with FTLBs (5, 6, 11, 23, 40, 43). Indeed, the combination of self-injury plus coprophenomena is highly specific to FTLBs relative to TS in several reports, though not diagnostic in isolation (11, 40, 43). Interestingly, two reports suggest that 50%–60% of patients with comorbid FTLBs and TS could distinguish their own FTLB symptoms from their typical tics of TS—suggesting the presence of intra-individual factors between the two symptoms (11, 12).

In addition to tics, many patients with TS report experiencing premonitory urges (32, 44) as well as the ability to temporarily suppress tic expression (45–47). However, the data on premonitory urges and suppressibility for FTLBs is mixed. Some reports suggest that individuals with FTLBs experience premonitory urges less frequently and have reduced suppressive abilities compared to individuals with TS (18, 21, 48, 49) whereas other reports suggest that individuals with FTLBs have a high level of premonitory urges and enhanced suppression abilities (23, 31, 34, 40). A study that specifically examined rates of premonitory urges and suppressive abilities directly compared individuals with TS versus FTLBs versus comorbid TS and FTLBs, and found no difference between the three groups (50). A different study found that 60% of patients with comorbid TS and FTLBs could subjectively distinguish between the two based on an absence of premonitory urge for FTLBs (11).

While FTLBs have a reliably acute onset, the clinical trajectory is variable. An international registry suggested that the median duration of symptoms was 12 months (mean=19 months; Range: 1 month—192 months), with most patients reporting that FTLBs began to peak in severity within a month (21). Based on the extant literature from a few prospective studies that had sample sizes ranging from 11 to 83 participants, three clinical trajectories emerged: 1) global symptom improvement including remission; 2) improvement in FTLBs with or without other significant impairment present; and 3) persistent FTLBs with significant impairment present–with or without worsening of symptoms (35, 51–53). Some instances of spontaneous remission were identified without active intervention, though in many cases when spontaneous remission was identified, it was later followed by re-emergence of FTLBs (21, 53). Notably, 40% of a retrospective cohort developed *additional* FND symptoms in between initial assessment and follow-up, which occurred an average of 2.6 years after FTLB onset (51). Several factors have been associated with persistence of FTLBs and dysfunction that include: 1) comorbid functional neurological symptoms (53), 2) higher baseline FTLB/tic severity (53), and 3) gender diversity status (25). Meanwhile, there are also prognostic indicators of FTLB symptom improvement that include: 1) a shorter time to an appropriate diagnosis (26, 52), 2) a younger age of symptom onset—prior to 18 years of age (23, 26, 52, 53), 3) male sex (52), and 4) discontinuation of secondary gain (52).

### Comorbid psychiatric conditions

Similar to TS (54, 55), co-occurring psychiatric conditions appear to be the rule rather than the exception for individuals with FTLBs—with an international registry identifying that up to 80% of patient with FTLBs have one or more co-occurring psychiatric conditions (21). Individuals with FTLBs exhibit high rates of anxiety disorders (27%–100%, with most cohorts exhibiting over 60% prevalence) (19, 21, 31, 48), and mood disorders (10%–72%) (4, 25, 36, 51). Neuropsychiatric conditions such as TS (15%–50%) (3, 23, 34, 35) and FND (16%–80%) (35, 40, 56) are also commonly comorbid (11, 40, 48, 53). Meanwhile, reports suggest that individuals with FTLBs variably present with co-occurring autism spectrum disorders (ASD; 0%–32%) (3, 11), obsessive compulsive disorder (OCD; 0%–42%) (17, 32), and attention deficit hyperactivity disorder (ADHD; 8%–42%) (17, 51) compared to controls and/or patients with TS only. Few reports identified prevalences of personality disorders (19%–46%) (23, 36) or eating disorders (6%–11%) (36, 52, 57). For those with comorbid TS and FTLBs, prevalence of psychiatric comorbidity was compared to those with TS only: one sample found higher rates of anxiety, high-functioning ASD, and ADHD (11), whereas another sample found higher rates of OCD but no differences in rates of ADHD, depression, and/or anxiety disorders (though this latter sample excluded social media-associated cases, and thus direct comparison is limited) (12).

### Types of treatment and response to interventions

For patients with TS, the two frontline treatments are behavioral interventions [e.g., Comprehensive Behavioral Intervention for Tics (CBIT)] (58–60) and pharmacotherapy (e.g., alpha agonists, antiseizure medication, antipsychotic medication) (61, 62). Only one report retrospectively examined whether CBIT improves FTLBs, finding it conferred no advantage over other psychotherapeutic modalities (25). Meanwhile, several reports retrospectively reported on evidence-based pharmacotherapy for TS applied to FTLBs, consistently showing that these pharmacological agents do not provide benefit to individuals with FTLBs (3, 21, 32, 52, 53). An international registry reported that out of 122 patients treated with clonidine, guanfacine, aripiprazole, or risperidone, 73%–89% had no improvement in FTLBs, and any benefit experienced by the minority of the sample was partial and transient (21).

When exploring evidence-based treatments that have shown therapeutic benefit in other mental health conditions, a different pattern potentially emerges, and small retrospective reports identify that CBT, serotonin reuptake inhibitors (SRIs), and/or the combination of the two treatments (CBT + SRI) may potentially have therapeutic benefit for patients with FTLBs, though interpretation is limited by study size, design, and quality. For example, 79% of patients in one series were retrospectively found to have improvement of FTLBs after receiving psychotherapy of various modalities (25), and a prospective report showed that receiving CBT for anxiety and/or depression was associated with lower tic severity at six month follow up (53). Some reports suggest that psychoeducation about FTLBs by itself can provide some symptomatic benefit (25, 34, 63). However, overall results on psychotherapies are mixed: a prospective cohort study found that neither the presence of therapy nor type of psychotherapy was associated with significant FTLB symptom improvement (52). A small uncontrolled case series proposed a new model of integrated cognitive behavioral therapy for functional tics (I-CBiT), and all eight individuals experienced FTLB symptom improvement, but some experienced decreased mood and emergence of other FND symptoms (64).

As for psychopharmacology, in one retrospective analysis, 78% of 46 patients treated with an SRI reported improvement in FTLBs (25), and a prospective cohort reported improvement (although not statistically significant) in FTLBs for those receiving SRI treatment at six-month follow up (53).

## Discussion

While the presence of functional neurological symptoms—and particularly FTLBs—is not a new phenomenon, there was an international rise in their occurrence in the context of the COVID-19 pandemic. This systematic narrative review of published reports sought to characterize information regarding the demographic characteristics, phenomenology, clinical course, comorbid conditions, and response to interventions of individuals with FTLBs. Though heterogeneity of both study methodology and the FTLB subtypes studied (e.g., *de novo*, functional overlay, and social-media-associated presentations) complicates direct comparison of some of these papers**,** several key findings were replicated across reports, and several gaps in the literature were identified.

First, regarding demographic characteristics, several factors were linked to FTLBs including adolescent age, female sex, and having a diverse gender identity—with gender identity suggested to be more strongly associated with onset of FTLBs than biological sex and corresponding with greater symptom severity. While these are promising descriptive insights, it also highlights several areas that warrant future research and consideration. For instance, given its primary onset in adolescence, it would be useful to determine whether biological age or pubertal development are more strongly linked to FTLBs.

Second, regarding psychosocial factors, both tics and FTLBs have evidence of spread via social contagion (e.g., children with TS exhibit echophenomena and often “pick-up” new tics after seeing others in TS camp, and adolescents can pick-up new FTLBs when viewing social media) (65). Indeed, several reports have strongly linked social media use to the emergence of new FTLBs (21, 23, 39). While stress and urge intolerance have been linked to tic expression in TS (46, 47), many individuals with FTLBs seem to have a greater sensitivity to stress—consistent with a stress-diathesis model whereby a provoking event can trigger an FND in someone who is predisposed (66). The clear provoking event (for a large percentage of the studies included in this review) was the COVID-19 global pandemic and its associated psychological sequelae (e.g., stress, social isolation), which theoretically contributed to increased social media usage and exposure to online tic content, and therefore may have created a positive feedback cycle of stress and FTLBs.

Several other relevant psychosocial factors remained absent from these investigations. While social media is implicated in FTLBs, there has been minimal examination of social networks or social identity in these contexts, and it is possible that understanding these relationships could provide unique insights regarding the pathogenesis and maintenance of FTLBs (e.g., if a social network engages in a certain behavior, it can get incorporated into social identity, and then the group will work to maintain the behavior in order to maintain the identity) (67). Similarly, there was no examination of associative learning or social learning, which have been implicated in the etiology, maintenance, and treatment of TS (29) and OCD (68, 69), and therefore it is possible they could play a role in symptom onset and/or maintenance of FTLB (e.g., if an individual with FTLBs has greater sensitivity to reward/reinforcement learning, then receiving positive social attention for the FTLBs could reinforce symptom expression and/or a specific identity of being a patient with FTLBs). Indeed, secondary gain—whether intentional or unintentional—was identified as a perpetuating factor of FTLBs across multiple studies (25, 52). Additionally, there was no investigation into the relationship between FTLBs and social determinants of health (e.g., economic stability, education, healthcare access/quality, neighborhood environment, etc.), which is highly relevant because these have been linked to disease chronicity (70), mental health (71), and quality of life (72) in other conditions.

Regarding phenomenology and clinical course, several key points were identified. First, while FTLBs are characterized by the acute onset of explosive complex motor movements and vocalizations, there are similarities in the tics between TS and FTLB patients with only a few movements/vocalizations more linked to FTLBs than TS (e.g., complex motor and vocal tics, coprolalia, and self-injurious behaviors) though none of them are diagnostic in isolation. Second, there is mixed evidence regarding whether FTLBs are associated with a premonitory urge and whether these symptoms can be suppressed. These mixed findings highlight the role of suggestibility and variability in the expression and experience of FTLBs. Recent theoretical work has proposed an integrative neuropsychological model of tic generation that frames the urge-action relationship in TS as a dynamic process involving interoceptive awareness, sensorimotor integration, top-down inhibitory control, and reinforcement learning (73). Although developed for neurodevelopmental tics, this framework may prove valuable for interpreting phenomenological differences and similarities between TS and FTLBs — particularly the variable presence and quality of premonitory sensations, and the differential capacity for symptom suppression across subgroups. Future research should leverage advanced statistical methods (e.g., latent profile analysis) to develop data-driven profiles that can account for these intra- and inter-individual differences, and could productively test whether the model's mechanistic components differ systematically between patients with TS, FTLBs, and mixed presentations.

Third, the available evidence suggests that there are approximately three distinct clinical courses of FTLB symptoms with only a few prognostic indicators of symptom severity. However, it is important to consider that many of the findings related to phenomenology and clinical course are retrospective in nature, with only a few prospective cohort studies, and none of those had a comparator group (35, 51–53). There is a clear need for future research using prospective designs with case-control comparisons to determine the risk factors associated with symptom onset and exacerbation, and to provide insights that will inform development of treatment initiatives. For instance, prospective monitoring of specific psychosocial stressors associated with FTLBs (e.g., social isolation, family conflict) may determine which features and severity level contribute most to symptom expression. Once identified, evidence-based interventions can be implemented to curtail symptom emergence in a preventative manner (e.g., implementation of CBT prior to psychosocial event that will likely elicit or worsen FTLBs). Finally, despite the improved characterization of phenomenology and clinical course, clear consensus diagnostic criteria to diagnose FTLBs remain absent from the field, though a growing body of evidence is validating guidelines from the European Society for the Study of Tourette Syndrome (11, 40, 74, 75). Consensus and broadly adopted diagnostic criteria are critical to advance the field in a systematic manner.

Regarding comorbid psychiatric conditions, there is consistent evidence that FTLBs frequently present alongside anxiety and depressive disorders, and many individuals with FTLBs also have TS or FND. While these are important findings that help identify potential shared factors underlying FTLBs, several key aspects remain unexamined. For instance, the time course of comorbid conditions was frequently not reported, therefore it is unclear whether FTLBs emerge before, concurrently with, or after comorbid conditions. Importantly, comorbidity ascertainment across the FTLB literature typically relied on clinical assessment rather than standardized psychiatric instruments, therefore limiting the reliability and cross-study comparability of comorbidity prevalence estimates. Additionally, while information about comorbid conditions is important, these classifications simplify heterogenous disorders into broad diagnostic categories, and greater utilization of dimensional approaches would be helpful to elucidate the shared and unique associations of FTLBs. Future research should build off work that highlights higher rates of insecure attachment, social anxiety, and borderline personality traits among individuals with FTLBs and conduct further investigation into these dimensional constructs of attachment, personality, and temperament (23, 36, 64).

Regarding interventions for FTLBs, multiple reports suggest that psychoeducation, psychotherapy, and SRIs may provide some symptom relief. While this may provide hope to individuals with FTLBs and their providers, several points warrant further consideration. First, interpretation is limited by an absence of randomized controlled trials. Second, there is limited information regarding which components of therapy were utilized in treatment, and which symptoms were specifically targeted (e.g., anxiety, depression, or FTLBs themselves). Third, it is unclear whether and to what extent family members were involved to generalize therapeutic skill implementation to home environments. Fourth, it is unknown to what extent these treatments improve functional outcomes and/or quality of life. Fifth, many individuals with FTLBs end up on multiple psychiatric medications, thus there is uncertainty regarding the specific pharmacological mechanisms that provide the most symptom relief to individuals with FTLBs. Finally, when reviewing the scant literature on intervention for FTLBs, it is important to note that these studies did not utilize a prospective or controlled design (76, 77). Thus, future research should leverage prospective clinical trial designs with comprehensive assessment approaches to characterize the impact of treatment on FTLBs and comorbid conditions.

In conclusion, there have been considerable advancements in understanding FTLBs, a condition that resides at the intersection of neurodevelopmental disorders and functional neurological disorders. However, more research is needed to clarify several aspects of FTLBs. First, it is important to understand factors that are associated with symptom onset with notable regard to biological sex and gender. Second, it is critical to further validate a consensus definition of and diagnostic criteria for FTLBs. Third, it will be essential to understand the underlying mechanisms and contextual factors (e.g., psychological, social, environmental) that influence FTLB symptoms and their exacerbation (78). Finally, it will be key to better understand the longitudinal course and response to interventions of FTLBs in a prospective and controlled manner. Given the complexity and multi-faceted nature, a multidisciplinary approach incorporating neurology, psychiatry, and psychosocial interventions will be important. When doing so, clinical trials should investigate connection of interventions to theorized underlying mechanisms in order to provide key insights to personalize treatment recommendations.

## Author's Note

Poster presented at the Functional Neurological Disorder Society Annual Conference; June 2026; Baltimore, MD.

## Data Availability

The original contributions presented in the study are included in the article/Supplementary Material, further inquiries can be directed to the corresponding author.
