# Supplementary material for: A systematic review to characterize functional tic-like behaviors
Source: Front Child Adolesc Psychiatry. 2026 Oct 2;5:1936806. doi: 10.3389/frcha.2026.1936806 (PMC13672511; doi:10.3389/frcha.2026.1936806)
Supplement: Supplementary file 1 [file Datasheet1.pdf]

JBI Critical Appraisal — Case Series (10 items) · Munn et al. 2020

Study (Author, Year)	Q1. Were there clear criteria for inclusion in the case series	Q2. Was the condition measured in a stan	Q3. Were valid methods used for identification of	Q4. Did the case series have consecutive inclusion of partic	Q5. Did the case series have complete inclusion	Q6. Was there clear reporting of the demographic	Q7. Was there clear reporti	Q8. Were the outcomes or follow up results of cases	Q9. Was there clear reporting of the pres	Q10. Was statistical analysis appropriate	Yes	Applicable	% Yes	Rating
Mejia & Jankovic 2005	Y	N	U	Y	N	Y	U	N	U	Y	4	10	40%	Low
Demartini 2014	Y	N	Y	Y	U	Y	Y	N	Y	Y	7	10	70%	High
Robinson & Hedderly 2016	Y	Y	Y	Y	Y	Y	Y	N	Y	Y	9	10	90%	High
Ganos 2016	Y	N	Y	U	Y	Y	Y	N	Y	U	6	10	60%	Moderate
Okkels 2023	Y	N	Y	Y	U	Y	Y	Y	Y	Y	8	10	80%	High
Cavanna 2022 (TS with functional	Y	Y	Y	Y	U	Y	Y	N	Y	Y	8	10	80%	High
Fremer 2022	Y	Y	Y	Y	N	Y	Y	Y	Y	Y	9	10	90%	High
Buts 2022	Y	Y	Y	Y	U	Y	Y	N	Y	Y	8	10	80%	High
Owen 2022	Y	N	Y	U	N	Y	Y	U	Y	N	5	10	50%	Moderate
Firestone 2023	Y	N	N	U	Y	Y	Y	N	Y	Y	6	10	60%	Moderate
Manbeck & Samanta 2025	Y	N	Y	U	U	Y	Y	Y	Y	Y	7	10	70%	High
Cavanna 2023 (New-onset, 105 cases)	Y	Y	Y	Y	Y	Y	Y	N	Y	Y	9	10	90%	High

JBI Critical Appraisal — Cohort Studies (11 items) · Moola et al. 2020

Study (Author, Year)	Q1. Were the two groups similar	Q2. Were exposures measured	Q3. Was the exposure measured in a valid	Q4. Were confounding factors iden	Q5. Were strategies to deal with conf	Q6. Were participants	Q7. Were the outcomes measur	Q8. Was the f	Q9. Was follow-up comp	Q10. Were strategies to add	Q11. Was appropriate statistical anal	Yes	Applicable	% Yes	Rating
Hartung 2026 (long-term follow-up)	NA	NA	Y	Y	U	Y	Y	Y	Y	U	Y	7	9	78%	High
Prato 2023 (6+12 mo FU)	NA	NA	Y	Y	U	Y	Y	Y	U	U	Y	6	9	67%	Moderate
Nilles 2024 (6 + 12 mo FU)	NA	NA	Y	Y	Y	Y	Y	Y	Y	N	Y	8	9	89%	High
Ducroizet 2025 (QI design)	NA	NA	Y	Y	N	Y	N	N	Y	N	U	4	9	44%	Low
Tomczak 2024 (retrospective)	NA	NA	N	Y	U	Y	Y	Y	Y	N	Y	6	9	67%	Moderate

JBI Critical Appraisal — Analytical Cross-Sectional Studies (8 items) · Barker et al. 2026 (revised)

Study (Author, Year)	Q1 (Selection). Were the criteria for inclusion in	Q2 (Selection). Were objective	Q3 (Exposure). Was the exposure meas	Q4 (Outcome). Were the outcomes measured in	Q5 (Confounding). Were confounding facto	Q6 (Confounding). Were strategie	Q7 (Statistical). Was appropriate stati	Q8 (Reporting). Were the study subjects	Yes	Applicable	% Yes	Rating
Cavanna 2023 (Expanding phenotypes)	Y	Y	Y	U	U	N	Y	Y	5	8	63%	Moderate
Hartung 2024 (prevalence)	Y	N	N	N	U	N	Y	Y	3	8	38%	Low
Trau 2022 (diagnostic checklist)	Y	Y	Y	Y	Y	U	Y	Y	7	8	88%	High
Martino 2023 (international registry)	Y	Y	Y	Y	Y	U	Y	Y	7	8	88%	High
Armstrong-Javors 2024	Y	Y	U	U	Y	N	Y	Y	5	8	63%	Moderate
Pringsheim 2021 (registry viewpoint)	Y	Y	Y	Y	Y	Y	Y	Y	8	8	100%	High
Nilles 2024 (Phenomenology)	Y	Y	Y	Y	Y	Y	Y	Y	8	8	100%	High
Szejko 2023	Y	Y	U	Y	Y	U	Y	Y	6	8	75%	High
Mathew 2023	Y	Y	Y	Y	Y	U	Y	Y	7	8	88%	High
Andersen 2023	Y	Y	U	U	Y	Y	Y	Y	6	8	75%	High
Fremer 2023	Y	Y	U	U	Y	U	Y	Y	5	8	63%	Moderate
Han 2022	Y	U	U	U	U	N	Y	Y	3	8	38%	Low
Pringsheim & Martino 2021	Y	Y	Y	Y	Y	N	Y	Y	7	8	88%	High
Müller-Vahl 2023	Y	Y	U	Y	U	N	U	Y	4	8	50%	Moderate
Larsh 2022	Y	U	Y	U	Y	Y	Y	Y	6	8	75%	High
Baizabal-Carvallo 2023	Y	Y	Y	Y	Y	N	Y	Y	7	8	88%	High
Berg 2024	Y	Y	Y	Y	Y	Y	Y	Y	8	8	####	High

JBI Critical Appraisal — Case-Control Studies (10 items) · Moola et al. 2020

Study (Author, Year)	Q1. Were the groups comparable other than	Q2. Were cases and controls matched	Q3. Were the same criteria used for identification	Q4. Was exposure measured	Q5. Was exposure measured in the sam	Q6. Were confounding factors	Q7. Were strategies to deal with conf	Q8. Were outcomes assessed	Q9. Was the exposure period of interest	Q10. Was appropriate statistic	Yes	Applicable	% Yes	Rating
Cavanna 2023 (Neurodevelopmental vs functional)	Y	Y	Y	U	Y	Y	Y	Y	Y	Y	**9**	**10**	**90%**	**High**
Cavanna 2025 (TS + FTLB phenotype)	Y	Y	Y	U	Y	Y	U	Y	Y	Y	**8**	**10**	**80%**	**High**
Paulus 2021	Y	Y	N	U	Y	Y	U	U	Y	Y	**6**	**10**	**60%**	**Moderate**

JBI Critical Appraisal — Quasi-Experimental Studies (9 items) · Barker et al. 2024 (revised)

Study (Author, Year)	Q1 (Temporal precedence). Is it clear in the study	Q2 (Selection/allocation). Was there a contr	Q3 (Confounding). Were participants inclu	Q4 (Intervention administration). Were the participants	Q5 (Outcome measurement). Wer	Q6 (Outcome measurement). Were the outco	Q7 (Outcome measurement). Were outcomes measu	Q8 (Retention). Was follow	Q9 (Statistical). Was appropriate stati	Yes	Applicable	% Yes	Rating
Duncan 2024	Y	N	NA	NA	Y	NA	Y	N	Y	4	6	67%	Moderate
Maxwell 2023 (I-CBiT)	Y	N	NA	NA	Y	NA	Y	N	Y	4	6	67%	Moderate
